# Xiaoyaosan formula augments adjuvant therapy and enhances postoperative breast cancer care

**DOI:** 10.3389/fphar.2024.1388646

**Published:** 2024-08-09

**Authors:** Chao Wang, Lianfang Yin

**Affiliations:** Department of Chinese Medicine, The First Affiliated Hospital of Bengbu Medical University, Bengbu, Anhui, China

**Keywords:** postoperative breast cancer, Xiaoyaosan, adjuvant chemotherapy, quality of life, psychological pressure

## Abstract

**Introduction:**

Xiaoyaosan (XYS), a traditional Chinese formula, not only has good antitumor effects but also attenuates distress, anorexia, and quality of life (QoL) by regulating neurology, the microbiota, immunology, and oxidative stress. This study aimed to assess the effect of XYS on QoL, psychological pressure, and spiritual well-being in breast cancer patients undergoing adjuvant chemotherapy.

**Methods:**

This prospective cohort study enrolled 176 postoperative breast cancer patients who received adjuvant chemotherapy combined with (n = 81) or without (n = 95) XYS for comparison. The Quality-of-Life Questionnaire Core-30 (QLQ-C30), Hospital Anxiety and Depression Scale (HADS), University of California Los Angeles (UCLA-LS), and Functional Assessment of Chronic Illness Therapy–Spiritual Well-being (FACIT–Sp) scores were evaluated before adjuvant chemotherapy (T_0_) and after the first (T_1_), second (T_2_), third (T_3_), and fourth cycles (T_4_) of adjuvant chemotherapy.

**Results:**

XYS improved the QLQ-C30 score at T_2_ (*p* = 0.043), T_3_ (*p* = 0.021), and T_4_ (*p* = 0.040) and the QLQ-C30 score at T_4_ (*p* = 0.027); moreover, XYS attenuated the QLQ-C30 score at T_2_ (*p* = 0.040), T_3_ (*p* = 0.023), and T_4_ (*p* = 0.027). Regarding distress, XYS reduced the HADS-anxiety score at T_2_ (*p* = 0.010), T_3_ (*p* = 0.025), and T_4_ (*p* = 0.019) and the HADS-defined anxiety score at T_3_ (*p* = 0.038). XYS also decreased the HADS-depression score at T_2_ (*p* = 0.016), T_3_ (*p* = 0.018), and T_4_ (*p* = 0.017) and the HADS-defined depression rate at T_2_ (*p* = 0.047), T_3_ (*p* = 0.012), and T_4_ (*p* = 0.013). In addition, XYS decreased the UCLA-LS at T_2_ (*p* = 0.023) but enhanced the FACIT-Sp at T_2_ (*p* = 0.029) and T_4_ (*p* = 0.026). Furthermore, after adjustment via propensity score matching, most of the significant findings remained.

**Discussion:**

The addition of XYS to adjuvant chemotherapy improved QoL, psychological health, and spiritual well-being in breast cancer patients.

## 1 Introduction

According to a recent global cancer statistical report, breast cancer ranks high among all cancer cases in terms of both incidence and mortality (Sung et al., 2021). Profiting from screening technology and national health literacy education, an increasing number of breast cancer patients are being diagnosed at an early stage and are able to receive mastectomy, with a good prognosis estimation (Yang S. et al., 2022a; Ren et al., 2022; Alkabban and Ferguson, 2023). However, a large proportion of patients are indicated to receive adjuvant chemotherapy with or without anti-HER2 agents based on the type of agent used after surgery, which has a considerable impact on patients’ quality of life (QoL), vasomotor symptoms, distress, social functions, and so on (Grimison and Stockler, 2007; Liu et al., 2022; Pereira et al., 2022). These factors make improving QoL, mental health, and spiritual well-being essential for postoperative breast cancer patients undergoing adjuvant chemotherapy (Krzyzanowska et al., 2021; He et al., 2022).

Xiaoyaosan (XYS) is a traditional Chinese formula commonly consisting of eight main components, Radix Bupleuri, Radix *Angelicae sinensis*, Radix Paeoniae alba, Rhizoma *Atractylodis macrocephala*, Poria, Rhizoma Zingiberis Recens, Radix Glycyrrhizae, and Herba Menthae, which can also be modified by the addition of other individual components (Hu et al., 2021). Multiple types of XYS have been shown to have good antitumor effects on several cancers, such as ovarian cancer (Li et al., 2021), colorectal cancer (Zhang et al., 2020), and especially breast cancer (Chen et al., 2012). Moreover, numerous studies have reported the excellent effects of XYS on depression, anxiety, anorexia, and QoL, which may be attributed to its regulation of the hypothalamic‒pituitary‒adrenal axis, neural and synaptic plasticity, neuronal loss, microbiota components, immunology, and oxidative stress, as well as its ability to modify several key biological pathways, including the PI3K/AKT, TLR4/NLRP3, RAGE, and JAK/STAT pathways (Li et al., 2017; Zhao et al., 2020; Zhou et al., 2021; Zhu et al., 2021; Chen et al., 2022; Yan et al., 2022; Zeng et al., 2022; Jiao et al., 2023). Recently, XYS has been applied along with adjuvant chemotherapy in breast cancer patients, which attenuates the postoperative complications and toxic reactions of chemotherapy and prolongs disease-free survival and overall survival to some degree (Wang et al., 2006; Du et al., 2015; Suo et al., 2018; Lu, 2019). However, its impact on QoL, psychological pressure, and spiritual well-being regarding adjuvant therapy in breast cancer patients has been less reported.

The current study compared adjuvant chemotherapy plus XYS versus adjuvant chemotherapy alone in postoperative breast cancer patients, aiming to investigate the effect of XYS on QoL, anxiety, depression, loneliness, and spiritual well-being in postoperative patients with breast cancer.

## 2 Methods

### 2.1 Subjects

This prospective cohort study enrolled 176 breast cancer patients who planned to receive adjuvant chemotherapy or adjuvant chemotherapy plus XYS between August 2020 and October 2022. The inclusion criteria were as follows: 1) histologically confirmed breast cancer; 2) aged older than 18 years; 3) scheduled for adjuvant chemotherapy or adjuvant chemotherapy plus XYS; 4) adequate hepatic, kidney, and bone marrow function according to the investigator; and 5) willing to cooperate with this study. The exclusion criteria were as follows: 1) had other primary cancers; 2) had malignant hematological diseases; 3) had distant metastases; 4) had allergies or physical intolerance to the study drugs; and 5) were pregnant women or nursing mothers. Approval for this study was granted via the Ethics Committee of The First Affiliated Hospital of Bengbu Medical University with the approval number 2020159. After explaining the details of the study to the patients, the investigator asked the patients whether they would participate in the study. The participants signed the consent form if they agreed to participate in this study.

### 2.2 Data collection and sample detection

Clinical characteristics, which included the age, marital status, employment status, education level, residence status, lesion site, histologic grade, and tumor–node–metastasis (TNM) stage, were collected for all subjects. In addition, estrogen receptor (ER), progesterone receptor (PR), human epidermal growth factor receptor 2 (HER-2), and Ki67 expression were detected in the tumor tissue by immunohistochemistry.

### 2.3 Treatment

Patients received adjuvant chemotherapy or adjuvant chemotherapy plus XYS. This study did not intervene in patients’ treatments, which were based on their disease status, their own wishes, or doctors’ suggestions. XYS was administered once a day, 30 min prior to a meal, for four cycles (3 weeks per cycle). The contents of XYS included bupleurum (10 g), white peony (15 g), Tuckahoe (15 g), Atractylodis Macrocephalae Rhizoma (10 g), *Angelica sinensis* (10 g), Radix glycyrrhizae preparata (10 g), *Zingiber officinale* (5 g), jujube (10 g), and Mentha (4 g) (Hu et al., 2021). Furthermore, the XYS regimen was adjusted according to the condition of the patients. In detail, patients with diarrhea received *Dioscorea opposita* Thunb. or *Lablab purpureus* (L.) Sweet; patients with poor sleep quality (such as insomnia) received Semen Ziziphi Spinosae. In addition, adjuvant chemotherapy included the following regimens: AC-T, AC-TH, AC, AC-THP, and AC + H. ‘A’ represents anthracyclines, ‘C’ represents cyclophosphamide, ‘T’ represents taxane, ‘H’ represents trastuzumab, and ‘P’ represents pertuzumab. Conventionally, four cycles of adjuvant chemotherapy (‘A,’ ‘C,’ and ‘T’) were administered (3 weeks per cycle). Patients with a HER2-positive status received 1 year of targeted therapy (‘H’ and ‘P’) in addition to adjuvant chemotherapy. The detailed regimen used was described in the guidelines of the Chinese Society of Clinical Oncology (CSCO) for breast cancer 2020 and is available at http://www.csco.org.cn/cn/index.aspx.

### 2.4 Questionnaires

The Quality-of-Life Questionnaire Core 30 (QLQ-C30) is a scale that contains 30 items. The first 28 items of the questionnaire used a four-point response scale ranging from 1 to 4. Items 29 and 30 were designed to evaluate global health status and quality of life (QoL), respectively, and used a response scale ranging from 1 to 7. All the raw data were transformed to a 0- to 100-point scale. Higher scores for the functional scales and global health status indicated better functioning and overall QoL, while a high score for the symptom scale represented a high level of symptom distress. The Cronbach’s α coefficient for this QLQ-C30 exceeded 0.70 (Tung et al., 2016). The Hospital Anxiety and Depression Scale (HADS) for anxiety (HADS-A) subscale comprises seven items with scores ranging from 0 to 3 for each item, and the total scores range from 0 to 21; the higher the score is, the more severe the anxiety. The HADS for depression (HADS-D) subscale comprises seven items with scores ranging from 0 to 3 for each item, and the total score ranges from 0 to 21; the higher the score is, the more severe the depression. A previous study reported good internal consistency for both the anxiety (0.71) and depression (0.67) subscales (Su et al., 2012). The University of California Los Angeles Loneliness Scale (UCLA-LS) scale contains 20 items (each item has a four-point frequency score ranging from 1 to 4), of which nine items are scored in a reverse order. The higher the total score is, the greater the degree of loneliness. The internal consistency of the UCLA-LS was good (Cronbach’s α coefficient was 0.94) (Liu and Guo, 2008). The Functional Assessment of Chronic Illness Therapy–Spiritual Wellbeing (FACIT-Sp) scale contains 12 items, which involves three dimensions. Each item was scored from 0 to 4 by the Likert 5 grading method. The Cronbach’s α coefficient for the FACIT-Sp subscales ranged from 0.711 to 0.920 (Liu et al., 2016). These questionnaires were administered in written form in Chinese.

### 2.5 Outcome measures

The QLQ-C30, HADS-A, HADS-D, UCLA-LS, and FACIT-Sp scores were used for evaluating quality of life, anxiety, depression, loneliness, and spiritual well-being, respectively (Aaronson et al., 1993; Russell, 1996; Peterman et al., 2002; Snaith, 2003). The above parameters were assessed before adjuvant chemotherapy (T_0_) and after the first (T_1_), second (T_2_), third (T_3_), and fourth cycles (T_4_) of adjuvant chemotherapy.

### 2.6 Statistics

SPSS (IBM, United States) version 26.0 was used for data processing. Student’s t-test, the Mann‒Whitney *U* test, the χ^2^ test, and Fisher’s exact test were utilized for comparison analyses. To adjust the imbalance of the characteristics between the two groups, propensity score analysis with the nearest neighbor matching method was used. The matched analysis included a comprehensive assessment of various factors, including the age, marital status, employment status, level of education, place of residence, lesion site, histologic grading, TNM stage, ER status, PR status, HER-2 status, and Ki67. Thus, the two study groups were matched at a 1:1 ratio. *p* < 0.05 indicated statistical significance.

## 3 Results

### 3.1 Patient characteristics

A total of 176 eligible patients were analyzed in the study, consisting of 81 patients in the adjuvant chemotherapy plus XYS group and 95 patients in the adjuvant chemotherapy group (Table 1). In the adjuvant chemotherapy plus XYS group, the mean age was 51.0 ± 10.3 years; 9.9%, 69.1%, and 21.0% of patients were at TNM stage I, II, and III, respectively; and 55.6%, 25.9%, 9.9%, 7.4%, and 1.2% of patients received AC-T, AC-TH, AC, AC-THP, and AC + H as adjuvant chemotherapy regimens, respectively. In the adjuvant chemotherapy group, the mean age was 51.8 ± 10.7 years; 14.7%, 60.0%, and 25.3% of patients were at TNM stage I, II, and III, respectively; and 60.0%, 18.9%, 9.5%, 9.5%, and 2.1% of patients received AC-T, AC-TH, AC, AC-THP, and AC + H as adjuvant chemotherapy regimens, respectively. In comparison, most of the patients’ characteristics did not differ between the two groups, but the level of education was greater in the adjuvant chemotherapy plus XYS group than in the adjuvant chemotherapy group (*p* = 0.020).

**TABLE 1:** Clinical characteristics of breast cancer patients.

Characteristic	Adjuvant chemotherapy (N = 95)	Adjuvant chemotherapy plus XYS (N = 81)	*p*-value
Age (years), mean ± SD	51.8 ± 10.7	51.0 ± 10.3	0.599
Married, No. (%)	84 (88.4)	66 (81.5)	0.196
Employed, No. (%)	45 (47.4)	44 (54.3)	0.358
Level of education, No. (%)			0.020
Primary school	8 (8.4)	2 (2.5)	
Middle school	28 (29.5)	14 (17.3)	
High school	40 (42.1)	44 (54.3)	
Undergraduate or above	19 (20.0)	21 (25.9)	
Residence, No. (%)			0.673
Rural	10 (10.5)	7 (8.6)	
Urban	85 (89.5)	74 (91.4)	
Lesion site, No. (%)			0.391
Unilateral	85 (89.5)	69 (85.2)	
Bilateral	10 (10.5)	12 (14.8)	
Histologic grading, No. (%)			0.084
Grade I	24 (25.3)	12 (14.8)	
Grade II	48 (50.5)	43 (53.1)	
Grade III	23 (24.2)	26 (32.1)	
T-stage, No. (%)			0.083
1	25 (26.3)	11 (13.6)	
2	58 (61.1)	58 (71.6)	
3	12 (12.6)	12 (14.8)	
N-stage, No. (%)			0.175
0	50 (52.6)	52 (64.2)	
1	24 (25.3)	14 (17.3)	
2	19 (20.0)	13 (16.0)	
3	2 (2.1)	2 (2.5)	
M-stage, No. (%)			1.000
0	95 (100.0)	81 (100.0)	
TNM stage, No. (%)			0.999
I	14 (14.7)	8 (9.9)	
II	57 (60.0)	56 (69.1)	
III	24 (25.3)	17 (21.0)	
ER positive, No. (%)	55 (57.9)	47 (58.0)	0.986
PR positive, No. (%)	52 (54.7)	41 (50.6)	0.585
HER-2 positive, No. (%)	29 (30.5)	28 (34.6)	0.568
Ki67 ≥ 30%, No. (%)	31 (32.6)	32 (39.5)	0.343
Adjuvant chemotherapy regimen, No. (%)			0.827
AC-T	57 (60.0)	45 (55.6)	
AC-TH	18 (18.9)	21 (25.9)	
AC	9 (9.5)	8 (9.9)	
AC-THP	9 (9.5)	6 (7.4)	
AC + H	2 (2.1)	1 (1.2)	

XYS, Xiaoyaosan; SD, standard deviation; TNM, tumor–node–metastasis; ER, estrogen receptor; PR, progesterone receptor; HER-2, human epidermal growth factor receptor 2; A, anthracycline; C, cyclophosphamide; T, taxane; H, trastuzumab; P, pertuzumab.

### 3.2 QoL comparison

**FIGURE 1:**
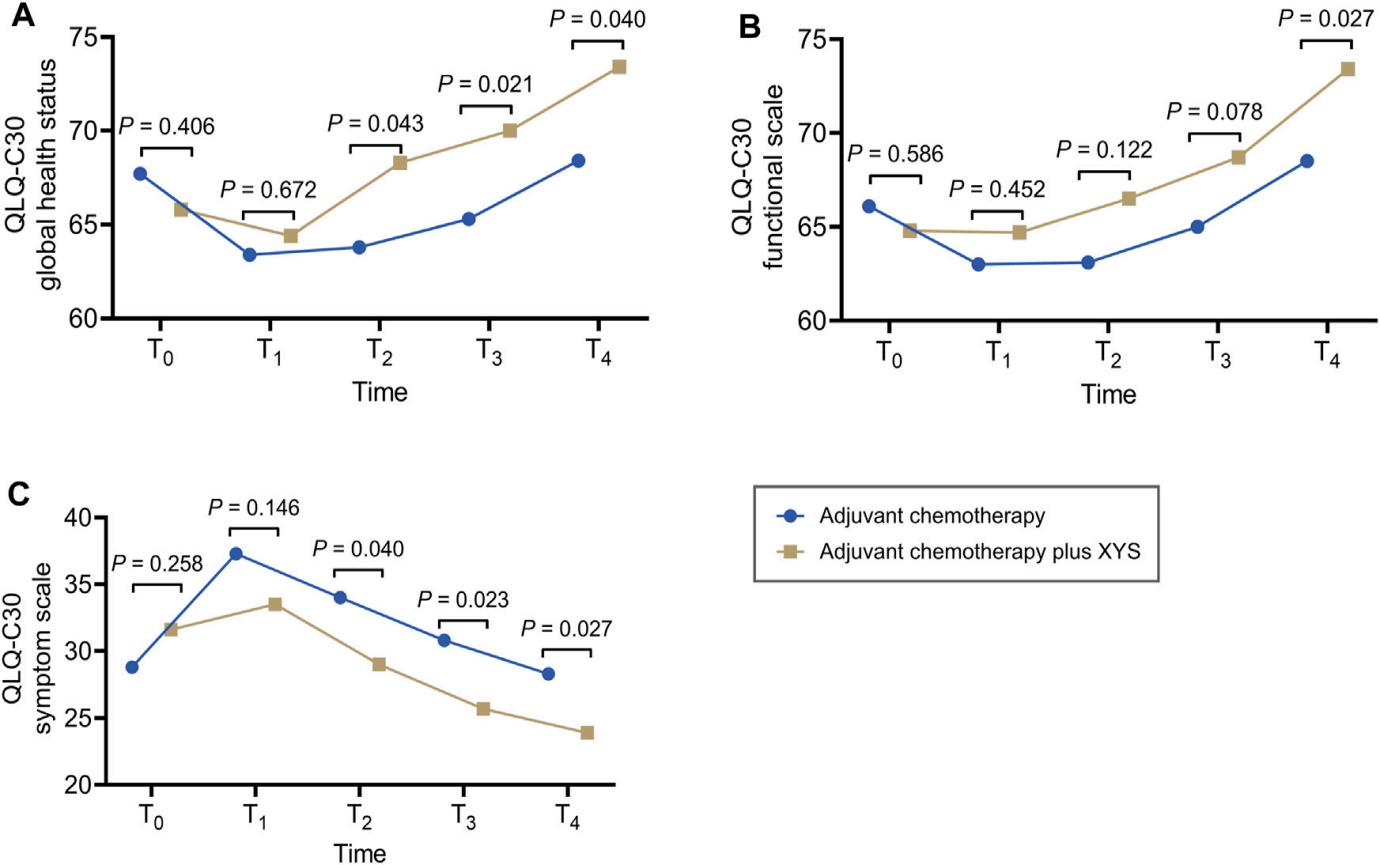
XYS improved QoL. Comparison of the QLQ-C30 global health status **(A)**, functional scale **(B)**, and symptom scale **(C)** at T_0_, T_1_, T_2_, T_3_, and T_4_ between the adjuvant chemotherapy plus XYS group and the adjuvant chemotherapy group.

Generally, in both the adjuvant chemotherapy plus XYS group and the adjuvant chemotherapy group, the QLQ-C30 global health status and functional scale showed a decreasing trend from T_0_ to T_1_ and then gradually increased thereafter to T_4_ (Figures 1A, B); the QLQ-C30 symptom scale revealed the opposite trend (Figure 1C).

In comparison, the QLQ-C30 global health status did not differ between T_0_ (*p* = 0.406) and T_1_ (*p* = 0.672) but was greater at T_2_ (*p* = 0.043), T_3_ (*p* = 0.021), and T_4_ (*p* = 0.040) in the adjuvant chemotherapy plus XYS group than in the adjuvant chemotherapy group (Figure 1A). The QLQ-C30 functional scale did not vary at T_0_ (*p* = 0.586), T_1_ (*p* = 0.452), T_2_ (*p* = 0.122), or T_3_ (*p* = 0.078) and was greater only at T_4_ (*p* = 0.027) in the adjuvant chemotherapy plus XYS group than in the adjuvant chemotherapy group (Figure 1B). In addition, the QLQ-C30 symptom scale did not differ at T_0_ (*p* = 0.258) or T_1_ (*p* = 0.146) but was lower at T_2_ (*p* = 0.040), T_3_ (*p* = 0.023), and T_4_ (*p* = 0.027) in the adjuvant chemotherapy plus XYS group than in the adjuvant chemotherapy group (Figure 1C).

### 3.3 Anxiety comparison

The HADS-A score did not differ at T_0_ (*p* = 0.902) or T_1_ (*p* = 0.146) but was lower at T_2_ (*p* = 0.010), T_3_ (*p* = 0.025), or T_4_ (*p* = 0.019) in the adjuvant chemotherapy plus XYS group than in the adjuvant chemotherapy group (Table 2). Moreover, the HADS-defined anxiety rate did not vary at T_0_ (*p* = 0.717), T_1_ (*p* = 0.275), T_2_ (*p* = 0.052), or T_4_ (*p* = 0.064) but was lower at T_3_ (*p* = 0.038) in the adjuvant chemotherapy plus XYS group than in the adjuvant chemotherapy group.

**TABLE 2:** Comparison of HADS-A scores and anxiety rates between the two groups.

Item	Adjuvant chemotherapy	Adjuvant chemotherapy plus XYS	*p*-value
HADS-A score, mean ± SD			
T_0_	7.7 ± 3.8	7.6 ± 3.1	0.902
T_1_	8.2 ± 2.9	7.6 ± 2.2	0.146
T_2_	8.2 ± 3.0	7.1 ± 2.2	0.010
T_3_	7.9 ± 3.0	6.9 ± 2.3	0.025
T_4_	7.7 ± 3.3	6.6 ± 2.6	0.019
Anxiety rate*, %			
T_0_	36.8	39.5	0.717
T_1_	46.2	37.8	0.275
T_2_	46.1	31.0	0.052
T_3_	43.7	27.5	0.038
T_4_	40.2	26.1	0.064

HADS-A, Hospital Anxiety and Depression Scale for anxiety; XYS, Xiaoyaosan; SD, standard deviation; T_0_, before adjuvant chemotherapy; T_1_, first cycle after adjuvant chemotherapy; T_2_, second cycle after adjuvant chemotherapy; T_3_, third cycle after adjuvant chemotherapy; T_4_, fourth cycle after adjuvant chemotherapy. ‘*’, a HADS-A score of more than 7 was defined as anxiety.

### 3.4 Depression comparison

The HADS-D score did not differ at T_0_ (*p* = 0.538) or T_1_ (*p* = 0.255) but was lower at T_2_ (*p* = 0.016), T_3_ (*p* = 0.018), or T_4_ (*p* = 0.017) in the adjuvant chemotherapy plus XYS group than in the adjuvant chemotherapy group (Table 3). Moreover, the HADS-defined depression rate did not differ at T_0_ (*p* = 0.485) or T_1_ (*p* = 0.393) but was lower at T_2_ (*p* = 0.047), T_3_ (*p* = 0.012), or T_4_ (*p* = 0.013) in the adjuvant chemotherapy plus XYS group than in the adjuvant chemotherapy group.

**TABLE 3:** Comparison of HADS-D scores and depression rates between the two groups.

Item	Adjuvant chemotherapy	Adjuvant chemotherapy plus XYS	*p*-value
HADS-D score, mean ± SD			
T_0_	7.6 ± 3.0	7.4 ± 2.9	0.538
T_1_	7.9 ± 2.8	7.4 ± 2.8	0.255
T_2_	8.1 ± 3.1	6.9 ± 3.0	0.016
T_3_	7.8 ± 2.4	6.9 ± 2.4	0.018
T_4_	7.7 ± 2.8	6.6 ± 2.6	0.017
Depression rate^*^, %			
T_0_	40.0	34.6	0.485
T_1_	43.0	36.5	0.393
T_2_	44.9	29.6	0.047
T_3_	47.1	27.5	0.012
T_4_	43.7	24.6	0.013

HADS-D, Hospital Anxiety and Depression Scale for depression; XYS, Xiaoyaosan; SD, standard deviation; T_0_, before adjuvant chemotherapy; T_1_, the first cycle after adjuvant chemotherapy; T_2_, the second cycle after adjuvant chemotherapy; T_3_, the third cycle after adjuvant chemotherapy; T_4_, the fourth cycle after adjuvant chemotherapy. ‘*’, a HADS-D score greater than 7 was defined as depression.

### 3.5 Loneliness and spiritual well-being comparison

**FIGURE 2:**
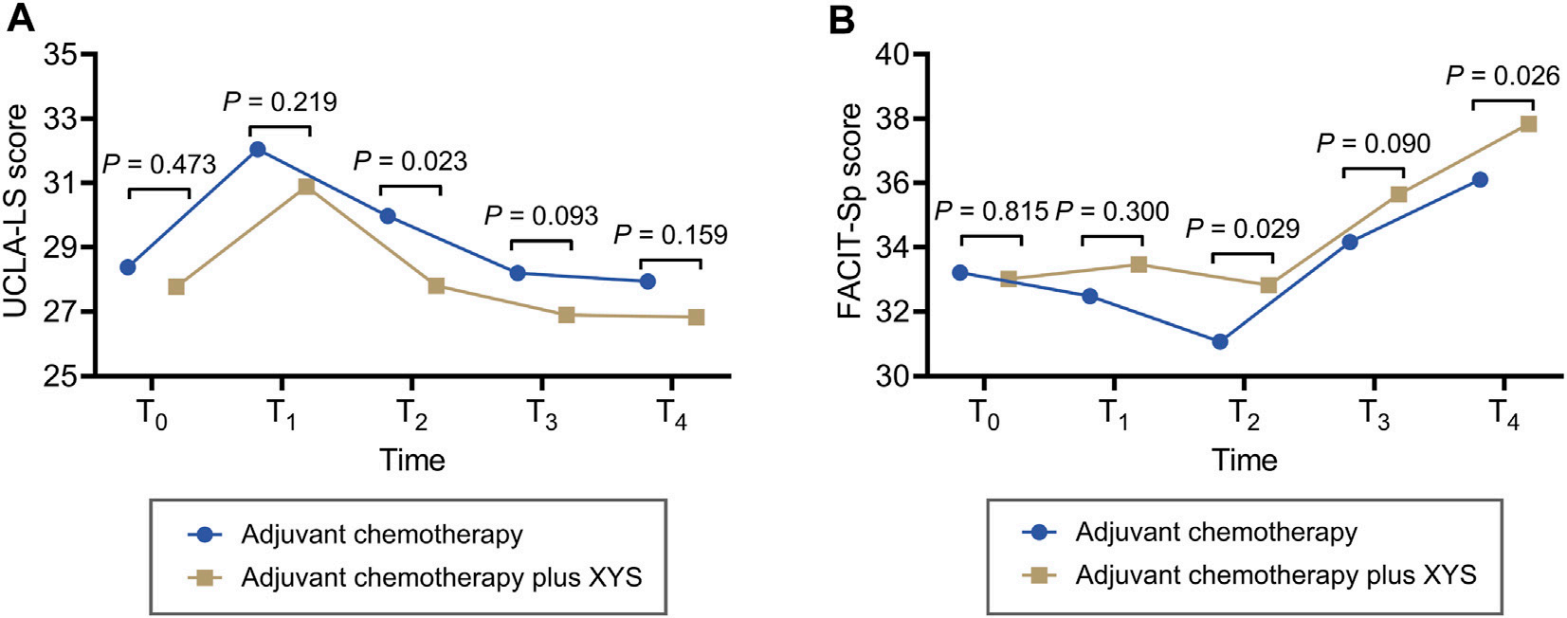
XYS attenuated loneliness and improved spiritual well-being. Comparisons of the UCLA-LS score **(A)** and FACIT-Sp score **(B)** at T_0_, T_1_, T_2_, T_3_, and T_4_ between the adjuvant chemotherapy plus XYS group and the adjuvant chemotherapy group.

UCLA-LS increased from T_0_ to T_1_ and then continuously decreased from T_1_ to T_4_ in both the adjuvant chemotherapy plus XYS group and the adjuvant chemotherapy group. In comparison, UCLA-LS was not different at T_0_ (*p* = 0.473), T_1_ (*p* = 0.219), T_3_ (*p* = 0.093), or T_4_ (*p* = 0.159) but was lower at T_2_ (*p* = 0.023) in the adjuvant chemotherapy plus XYS group than in the adjuvant chemotherapy group (Figure 2A).

FACIT-Sp did not fluctuate obviously from T_0_ to T_2_ and then greatly increased from T_2_ to T_4_ in the adjuvant chemotherapy plus XYS group; however, it decreased from T_0_ to T_2_ first and then gradually increased from T_2_ to T_4_ in the adjuvant chemotherapy group. In comparison, FACIT-Sp did not vary at T_0_ (*p* = 0.815), T_1_ (*p* = 0.300), or T_3_ (*p* = 0.090) but was greater at T_2_ (*p* = 0.029) and T_4_ (*p* = 0.026) in the adjuvant chemotherapy plus XYS group than in the adjuvant chemotherapy group (Figure 2B).

### 3.6 Findings after the adjustment by propensity score matching

Considering that some of the characteristics differed between the adjuvant chemotherapy plus XYS group and the adjuvant chemotherapy group, propensity score matching was performed for adjustment. All of the characteristics did not differ between the two groups after adjustment by propensity score matching (Table 4).

**TABLE 4:** Clinical characteristics of breast cancer patients after adjustment via propensity score analysis.

Characteristic	Adjuvant chemotherapy ^ad^ (N = 73)	Adjuvant chemotherapy plus XYS ^ad^ (N = 73)	*P* ^*ad*^ value
Age (years), mean ± SD	52.9 ± 10.5	50.9 ± 10.4	0.253
Married, No. (%)	64 (87.7)	63 (86.3)	0.806
Employed, No. (%)	34 (46.6)	40 (54.8)	0.321
Level of education, No. (%)			0.299
Primary school	5 (6.8)	2 (2.7)	
Middle school	20 (27.4)	13 (17.8)	
High school	31 (42.5)	43 (58.9)	
Undergraduate or above	17 (23.3)	15 (20.5)	
Residence, No. (%)			0.574
Rural	8 (11.0)	6 (8.2)	
Urban	65 (89.0)	67 (91.8)	
Lesion site, No. (%)			0.796
Unilateral	65 (89.0)	64 (87.7)	
Bilateral	8 (11.0)	9 (12.3)	
Histologic grading, No. (%)			0.577
Grade I	16 (21.9)	12 (16.4)	
Grade II	35 (47.9)	38 (52.1)	
Grade III	22 (30.1)	23 (31.5)	
T-stage, No. (%)			0.291
1	19 (26.0)	11 (15.1)	
2	44 (60.3)	53 (72.6)	
3	10 (13.7)	9 (12.3)	
N-stage, No. (%)			0.178
0	39 (53.4)	47 (64.4)	
1	17 (23.3)	14 (19.2)	
2	15 (20.5)	10 (13.7)	
3	2 (2.7)	2 (2.7)	
M-stage, No. (%)			1.000
0	73 (100.0)	73 (100.0)	
TNM stage, No. (%)			0.442
I	9 (12.3)	8 (11.0)	
II	44 (60.3)	51 (69.9)	
III	20 (27.4)	14 (19.2)	
ER positive, No. (%)	42 (57.5)	41 (56.2)	0.867
PR positive, No. (%)	39 (53.4)	38 (52.1)	0.868
HER-2 positive, No. (%)	22 (30.1)	24 (32.9)	0.722
Ki67 ≥ 30%, No. (%)	24 (32.9)	28 (38.4)	0.489
Adjuvant chemotherapy regimen, No. (%)			0.754
AC-T	46 (63.0)	41 (56.2)	
AC-TH	15 (20.5)	19 (26.0)	
AC	5 (6.8)	8 (11.0)	
AC-THP	5 (6.8)	4 (5.5)	
AC + H	2 (2.7)	1 (1.4)	

XYS, Xiaoyaosan; SD, standard deviation; TNM, tumor–node–metastasis; ER, estrogen receptor; PR, progesterone receptor; HER-2, human epidermal growth factor receptor 2; T_0_, before adjuvant chemotherapy; T_1_, the first cycle after adjuvant chemotherapy; T_2_, the second cycle after adjuvant chemotherapy; T_3_, the third cycle after adjuvant chemotherapy; T_4_, the fourth cycle after adjuvant chemotherapy. ‘ad’ indicates that the data were adjusted via propensity score analysis.

After adjustment, the QLQ-C30 global health status (*p* = 0.991), functional scale score (*p* = 0.909), symptom scale score (*p* = 0.645), HADS-A score (*p* = 0.700), HADS-defined anxiety rate (*p* = 0.389), HADS-D score (*p* = 0.749), HADS-defined depression rate (*p* = 1.000), UCLA-LS score (*p* = 0.570), and FACIT-Sp score (*p* = 0.558) were not different at T_0_ between the adjuvant chemotherapy plus XYS group and the adjuvant chemotherapy group. Importantly, the QLQ-C30 global health status (*p* = 0.013), QLQ-C30 functional scale score (*p* = 0.010), and FACIT-Sp score (*p* = 0.035) were greater at T_4_ in the adjuvant chemotherapy plus XYS group than in the adjuvant chemotherapy group. Moreover, the HADS-D (*p* = 0.002) and HADS-defined depression rates (*p* = 0.010) were lower at T_4_ in the adjuvant chemotherapy plus XYS group than in the adjuvant chemotherapy group (Table 5). Furthermore, no liver- or kidney-related adverse events occurred in this study.

**TABLE 5:** Comparisons of the QLQ-C30 scores, HADS-A scores, anxiety rates, HADS-D scores, depression rates, UCLA-LS scores, and FACIT-Sp scores between the two groups after adjustment.

Item	Adjuvant chemotherapy ^ad^	Adjuvant chemotherapy plus XYS ^ad^	*P* ^*ad*^ value
QLQ-C30 global health status, mean ± SD			
T_0_	67.3 ± 13.6	67.3 ± 15.9	0.991
T_4_	67.9 ± 14.8	74.5 ± 15.3	0.013
QLQ-C30 functional scale, mean ± SD			
T_0_	65.5 ± 16.1	65.8 ± 15.7	0.909
T_4_	68.2 ± 13.7	74.3 ± 12.9	0.010
QLQ-C30 symptom scale, mean ± SD			
T_0_	29.4 ± 15.8	30.6 ± 16.5	0.645
T_4_	27.8 ± 13.0	23.7 ± 11.9	0.066
HADS-A score, mean ± SD			
T_0_	7.4 ± 3.7	7.6 ± 3.1	0.700
T_4_	7.7 ± 3.4	6.8 ± 2.6	0.077
Anxiety rate, %			
T_0_	32.9	39.7	0.389
T_4_	39.7	29.0	0.201
HADS-D score, mean ± SD			
T_0_	7.5 ± 2.9	7.3 ± 2.8	0.749
T_4_	7.8 ± 2.8	6.4 ± 2.4	0.002
Depression rate, %			
T_0_	35.6	35.6	1.000
T_4_	44.1	22.6	0.010
UCLA-LS score, mean ± SD			
T_0_	28.3 ± 6.3	27.7 ± 4.9	0.570
T_4_	27.7 ± 5.6	26.8 ± 3.5	0.271
FACIT-Sp score, mean ± SD			
T_0_	32.8 ± 5.3	33.4 ± 5.7	0.558
T_4_	36.1 ± 4.4	37.9 ± 5.0	0.035

QLQ-C30, Quality of Life Questionnaire Core 30; HADS-A, Hospital Anxiety and Depression Scale for anxiety; HADS-D, Hospital Anxiety and Depression Scale for depression; UCLA-LS, University of California Los Angeles Loneliness Scale; FACIT-Sp, Functional Assessment of Chronic Illness Therapy–Spiritual Well-being; XYS, Xiaoyaosan; SD, standard deviation; T_0_, before adjuvant chemotherapy; T_4_, the fourth cycle after adjuvant chemotherapy. ‘ad’ indicates that the data were adjusted via propensity score analysis. Anxiety was defined as a HADS-A score greater than 7, and depression was defined as a HADS-D score greater than 7.

### 3.7 Subgroup analysis

The subgroup analysis was carried out based on the TNM stage. These findings indicated that most outcomes were not statistically significant in the subgroup analysis, even though a tendency still existed. Specifically, the QLQ-C30 global health status score, QLQ-C30 functional scale score, and FACIT-Sp score were numerically greater, while the QLQ-C30 symptom scale score, HADS-A score, anxiety rate, HADS-D score, depression rate, and UCLA-LS score were numerically lower at T4 in the adjuvant chemotherapy plus XYS subgroup than in the adjuvant chemotherapy subgroup in the TNM stage I, II, and III subgroups (Supplementary Table S1).

## 4 Discussion

Previous studies have focused mainly on the ability of XYS to alleviate anxiety or depression, and some studies have explored its potential mechanism (Wang et al., 2024; Wu et al., 2024). For instance, one study indicated that XYS might inhibit neuroinflammation to treat depression by regulating the TRIM31/NLRP3 inflammasome (Wang et al., 2024). However, the effect of XYS on QoL in breast cancer patients has seldom been reported. In this study, the addition of XYS to chemotherapy might improve the QLQ-C30 global health and symptom scores. These findings were similar to those of the previous studies, which also reported that XYS might alleviate depression symptoms in patients with depression (Hu et al., 2021; Liu et al., 2021). Even though this study did not carry out any mechanistic experiment, the mechanism of XYS on improving the QLQ-C30 global health and symptom scores might be explained by referring to the previous study, which reported that XYS could relieve depression and anxiety through several mechanisms, such as participating in the autophagy of hypothalamic neurons (Peterman et al., 2002; Zeng et al., 2022; Wang et al., 2024), regulating synapses or synaptic-associated signals (Wu et al., 2024), regulating neurotransmitters (Hagiwara et al., 2023; Javan Biparva et al., 2023; Gudhoor et al., 2024), and regulating the brain–gut axis (Chen et al., 2022), thereby alleviating mental disorders to further improve the global health status and reduce the symptoms of breast cancer patients. However, these hypotheses about the mechanism still need further exploration. However, the functional score of the QLQ-C30 improved relatively little, which was mainly because the QLQ-C30 functional score mainly consisted of the physical function, role function, emotional function, cognitive function, and social function ((Hagiwara et al., 2023; Biparva et al., 2023)); XYS could only have a specific effect on emotional function and social function but had little impact on physical function, role function, or cognitive function. Therefore, chemotherapy plus XYS improved the QLQ-C30 functional score only to a certain degree (the significance of the difference between the two groups was observed only at T_4_).

Mental disorders, including anxiety and depression, are frequently reported in cancer patients, especially in breast cancer patients undergoing adjuvant chemotherapy, who might suffer from adverse reactions to mastectomy and chemotherapy (Hashemi et al., 2020; Biparva et al., 2023). It has been reported that the depression and anxiety rates in breast cancer patients are as high as 30.2% and 41.9%, respectively (Hashemi et al., 2020; Biparva et al., 2023). These mental disorders further induce low compliance with subsequent therapy and even lead to suicide (Bui and Fava, 2017). Hence, it is essential to relieve mental illness in patients with breast cancer. XYS is a traditional Chinese medicine consisting of *Angelicae Sinensis* Radix, Bupleuri Radix, Paeoniae Radix Alba, Atractylodis Macrocephalae Rhizoma, Poria, Zingiberis Rhizoma Recens, Glycyrrhizae Radix et Rhizoma, and Menthae Haplocalycis Herba, which can alleviate depression-like behavior by regulating necroptosis-related cellular senescence in the hypothalamus in a mouse model (Jiao et al., 2023). However, its clinical application for relieving mental disorders in breast cancer patients is scarce. In this study, treatment with adjuvant chemotherapy plus XYS decreased the depression rate from 34.6% to 24.6% and the anxiety rate from 39.5% to 26.1% in breast cancer patients, which was more satisfactory than that of adjuvant chemotherapy monotherapy. These findings could be explained as follows: (Sung et al., 2021): XYS might have an antidepressant effect through various mechanisms, such as downregulating A2AR signaling, participating in the autophagy of hypothalamic neurons by regulating GLUT4 expression, and promoting hippocampal neurogenesis (Yang FR. et al., 2022; Zeng et al., 2022; Zhu et al., 2022; Ren et al., 2022). XYS can also regulate synapse- or synaptic-associated signaling pathways, including the neurotrophin signaling and PI3K/AKT signaling pathways, thereby alleviating depression (Meng et al., 2023; Yang S. et al., 2022). XYS has a specific effect on regulating neurotransmitters such as dopamine, 5-hydroxytryptophan, and thyroid hormone; the latter plays an essential role in the pathogenesis of depression and anxiety (Kong et al., 2010; Ding et al., 2014; Zhang et al., 2021; Alkabban and Ferguson, 2023). XYS can also regulate the brain–gut axis, neuroinflammation, and the neuroendocrine axis, thus further acting on its antidepressant effect (Chen et al., 2022). Hence, XYS might play an antidepressant role in breast cancer patients in this study.

Additionally, XYS improved loneliness (as reflected by the UCLA-LS score) and spiritual well-being (as reflected by the FACIT-Sp score) in this study. These findings could be explained as follows: loneliness was positively associated with depression symptoms (Friedman-Ezra et al., 2024). XYS might alleviate depression symptoms through several mechanisms, as mentioned above; therefore, feelings of loneliness are relieved accordingly. Therefore, spiritual well-being is a subjective feeling based on comprehensive assessments of the overall status of the patients themselves. When the QoL improved, anxiety and depression were relieved, and their spiritual well-being improved accordingly.

There was an imbalance in the baseline (T_0_) characteristics between the two groups. Hence, the adjustment of the baseline characteristics via propensity score methods was carried out to eliminate this potential confounder (D Agostino, 1998). After the adjustment, there was no difference in the baseline characteristics between the two groups, and the difference in primary outcomes, including anxiety, depression, QoL, loneliness, and spiritual well-being, did not change between the two groups. These findings further validated the effectiveness of chemotherapy plus XYS in breast cancer patients.

Although some methods, such as the propensity score method, have been used for remedying methodological deficiencies, our study has several limitations (Sung et al., 2021). The sample size was small, and this limitation was aggravated after adjustment by the propensity score method. Hence, further studies with larger sample sizes are needed (Ren et al., 2022). The follow-up period should be prolonged due to the short follow-up period in this study (Yang S. et al., 2022). The TNM stage was mainly I–II in most patients (approximately 70%) in this study, indicating a relatively low disease burden. However, treatment strategies differ between early-stage and advanced-stage breast cancer patients, which means that the outcomes and treatment-related side effects also differ. Hence, further studies should be conducted to determine the efficacy of chemotherapy plus XYS in breast cancer patients with greater disease burdens, such as those with advanced breast cancer (Alkabban and Ferguson, 2023). When we would like to apply XYS in treating breast cancer patients, some research gaps needed to be solved, such as the detailed mechanism, its efficacy and safety, how to be involved in the current treatment strategy, treatment timing, dosage, and individualized treatment. Our study only preliminarily explored its efficacy and safety. However, more studies are needed to resolve other research gaps before extensively applying XYS in treating breast cancer patients.

In conclusion, adding XYS to adjuvant chemotherapy improved QoL, psychological pressure, and spiritual well-being in postoperative patients with breast cancer, and these advancements remained even after adjustment via propensity score analysis.

## Data Availability

The original contributions presented in the study are included in the article/Supplementary Material; further inquiries can be directed to the corresponding author.
