# Supplementary material for: Xiaoyaosan formula augments adjuvant therapy and enhances postoperative breast cancer care
Source: Front Pharmacol. 2024 Aug 9;15:1388646. doi: 10.3389/fphar.2024.1388646 (PMC11344260; doi:10.3389/fphar.2024.1388646)
Supplement: Supplementary file 1 [file Table1.DOCX]

***Supplementary Materials***

**Supplementary Table 1.** Subgroup analyses on comparison of QLQ-C30 scores, HADS-A scores, anxiety rates, HADS-D scores, depression rates, UCLA-LS scores, and FACIT-Sp scores between two groups.

| Items | TNM stage I | | | TNM stage II | | | TNM stage III | | |
| --- | --- | --- | --- | --- | --- | --- | --- | --- | --- |
|  | Adjuvant chemotherapy | Adjuvant chemotherapy plus XYS | *P* value | Adjuvant chemotherapy | Adjuvant chemotherapy plus XYS | *P* value | Adjuvant chemotherapy | Adjuvant chemotherapy plus XYS | *P* value |
| QLQ-C30 global health status, mean±SD |  |  |  |  |  |  |  |  |  |
| T0 | 69.8±15.2 | 59.5±15.6 | 0.145 | 68.4±13.7 | 64.8±16.3 | 0.202 | 64.6±12.1 | 72.1±14.7 | 0.082 |
| T4 | 73.0±14.8 | 67.9±17.7 | 0.496 | 67.3±14.9 | 72.8±15.6 | 0.082 | 68.1±14.6 | 77.5±12.9 | 0.045 |
| QLQ-C30 functional scale, mean±SD |  |  |  |  |  |  |  |  |  |
| T0 | 68.3±15.4 | 56.6±10.3 | 0.072 | 65.6±16.6 | 64.4±16.2 | 0.693 | 66.1±15.0 | 70.2±14.5 | 0.392 |
| T4 | 67.6±14.6 | 71.6±9.7 | 0.530 | 68.8±13.1 | 72.9±14.2 | 0.141 | 68.6±13.7 | 75.5±12.3 | 0.116 |
| QLQ-C30 symptom scale, mean±SD |  |  |  |  |  |  |  |  |  |
| T0 | 23.4±15.8 | 40.0±14.8 | 0.025 | 29.2±15.7 | 32.1±16.8 | 0.333 | 31.3±15.4 | 25.8±14.8 | 0.265 |
| T4 | 26.0±14.5 | 25.1±12.1 | 0.857 | 29.3±12.5 | 24.0±12.5 | 0.039 | 27.4±12.3 | 23.0±9.8 | 0.243 |
| HADS-A score, mean±SD |  |  |  |  |  |  |  |  |  |
| T0 | 8.1±4.2 | 6.9±3.3 | 0.497 | 7.2±3.3 | 7.8±3.3 | 0.324 | 8.7±4.6 | 7.5±2.6 | 0.324 |
| T4 | 6.6±1.9 | 6.4±3.5 | 0.876 | 7.7±3.5 | 6.6±2.5 | 0.076 | 8.4±3.4 | 6.6±2.6 | 0.078 |
| Anxiety rate, % |  |  |  |  |  |  |  |  |  |
| T0 | 50.0 | 37.5 | 0.675 | 33.3 | 41.1 | 0.395 | 37.5 | 35.3 | 0.885 |
| T4 | 23.1 | 28.6 | >0.999 | 39.2 | 23.9 | 0.107 | 52.2 | 31.3 | 0.195 |
| HADS-D score, mean±SD |  |  |  |  |  |  |  |  |  |
| T0 | 7.1±2.4 | 6.0±2.7 | 0.312 | 7.5±2.9 | 7.5±2.9 | 0.987 | 8.2±3.6 | 7.4±3.0 | 0.484 |
| T4 | 6.6±1.6 | 6.4±2.0 | 0.819 | 7.7±3.2 | 6.5±2.5 | 0.052 | 8.2±2.5 | 6.9±3.3 | 0.154 |
| Depression rate, % |  |  |  |  |  |  |  |  |  |
| T0 | 28.6 | 37.5 | >0.999 | 38.6 | 37.5 | 0.904 | 50.0 | 23.5 | 0.087 |
| T4 | 23.1 | 28.6 | >0.999 | 45.1 | 23.9 | 0.029 | 52.2 | 25.0 | 0.090 |
| UCLA-LS score, mean±SD |  |  |  |  |  |  |  |  |  |
| T0 | 28.5±6.8 | 26.5±1.8 | 0.428 | 27.4±4.9 | 27.8±5.4 | 0.681 | 30.5±8.1 | 28.1±4.1 | 0.263 |
| T4 | 28.3±6.7 | 26.4±2.1 | 0.486 | 27.2±4.5 | 26.7±4.0 | 0.579 | 29.5±7.1 | 27.4±3.6 | 0.297 |
| FACIT-Sp score, mean±SD |  |  |  |  |  |  |  |  |  |
| T0 | 32.8±5.2 | 28.4±3.9 | 0.049 | 33.6±5.4 | 33.2±5.6 | 0.675 | 32.5±5.7 | 34.6±6.2 | 0.264 |
| T4 | 35.5±4.3 | 35.4±6.2 | 0.963 | 36.2±5.0 | 37.9±4.9 | 0.089 | 36.3±4.4 | 38.8±4.5 | 0.096 |

QLQ-C30, Quality of Life Questionnaire Core 30; HADS-A, hospital anxiety and depression scale for anxiety; HADS-D, hospital anxiety and depression scale for depression; UCLA-LS, the University of California Los Angeles loneliness scale; FACIT-Sp, the functional assessment of chronic illness therapy-spiritual well-being; XYS, Xiaoyaosan; TNM, tumor-node- tumor-node-metastasis; SD, standard deviation; T0, before adjuvant chemotherapy; T4, the 4th cycle after adjuvant chemotherapy. ‘ad’ indicated that data was adjusted via propensity score analysis. Anxiety was defined as a HADS-A score of more than 7, and depression was defined as a HADS-D score of more than 7.
